# Increased nutritional value in food crops

**DOI:** 10.1111/1751-7915.12764

**Published:** 2017-07-11

**Authors:** Nieves Goicoechea, M. Carmen Antolín

**Affiliations:** ^1^ Universidad de Navarra Facultades de Ciencias y Farmacia y Nutrición Grupo de Fisiología del Estrés en Plantas (Departamento de Biología Ambiental) Unidad Asociada al CSIC (EEAD, Zaragoza, ICVV, Logroño) c/Irunlarrea 1 31008 Pamplona Spain

## Abstract

Modern agriculture and horticulture must combine two objectives that seem to be almost mutually exclusive: to satisfy the nutritional needs of an increasing human population and to minimize the negative impact on the environment. These two objectives are included in the Goal 2 of the 2030 Agenda for Sustainable Development of the United Nations: ‘End hunger, achieve food security and improved nutrition and promote sustainable agriculture'. Enhancing the nutritional levels of vegetables would improve nutrient intake without requiring an increase in consumption. In this context, the use of beneficial rhizospheric microorganisms for improving, not only growth and yield, but also the nutrient quality of crops represents a promising tool that may respond to the challenges for modern agriculture and horticulture and represents an alternative to the genetic engineering of crops. This paper summarizes the state of the art, the current difficulties associated to the use of rhizospheric microorganisms as enhancers of the nutritional quality of food crops as well as the future prospects.

## Abstract 2

This paper summarizes the state of the art, the current difficulties associated to the use of rhizospheric microorganisms as enhancers of the nutritional quality of food crops as well as the future prospects in the context of the Goal 2 of the 2030 Agenda for Sustainable Development of the United Nations: ‘End hunger, achieve food security and improved nutrition and promote sustainable agriculture'.

## Introduction

Plants are traditionally part of the human diet containing bioactive components that may exert physiological effects beyond nutrition promoting human health and well‐being. Regular consumption of fruits and vegetables is associated with reduced risks of chronic diseases such as cancer, cardiovascular disease, stroke, Alzheimer's disease, cataract and age‐related functional decline. To satisfy the food needs of the global population, farmers of all countries implemented the green revolution technology, which has caused deleterious effects on the environment and which also represents a latent problem for human health (Baez‐Rogelio *et al*., 2017). For that reason, modern agriculture and horticulture must combine two objectives that seem to be almost mutually exclusive: to satisfy the nutritional needs of an increasing human population and to minimize the negative impact on the environment (Duhamel and Vandenkoornhuyse, 2013). These two objectives are included in the Goal 2 of the 2030 Agenda for Sustainable Development of the United Nations: ‘End hunger, achieve food security and improved nutrition and promote sustainable agriculture'. Enhancing the nutritional levels of vegetables would improve nutrient intake without requiring an increase in consumption. Genetic engineering offers opportunities to significantly raise the nutritional levels of crops, but the commercialization of transgenic plants still awaits progress in transgene expression, public acceptance, intellectual property issues and risk assessment. There are also serious economic and marketing challenges to be overcome. In this context, the use of beneficial rhizospheric microorganisms for improving not only growth and yield but also the nutrient quality of crops represents a promising tool that may respond to the challenges for modern agriculture and horticulture.

The majority of studies dealing with the microbial communities colonizing the rhizosphere have focused on bacteria and fungi. Plant growth‐promoting rhizobacteria (PGPR) are a group of free‐living bacteria that promote plant growth by colonizing the roots of host plants and include bacteria belonging to the genera *Rhizobium, Brevibacillus, Pseudomonas, Pantoea, Serratia, Azospirillum* and *Bacillus*, among others. PGPR produce plant growth‐promoting compounds such as hormones, siderophores and antibacterial peptides, with the auxin production being one of the most widely used traits to select bacteria with potential plant growth‐promoting activity. PGPR have shown good effectiveness in terms of biofertilization, biocontrol and bioremediation and also play an important role in the adaptation of plants to the environment (Ruzzi and Aroca, 2015; Vejan *et al*., 2016). Therefore, PGPR may play an important role in the context of a sustainable agricultural industry because they allow increased crop production with significant reductions in synthetic chemical fertilizers and pesticides. Apart from enhancing plant growth and yield, PGPR may also improve the nutritional quality of fruits and vegetables. Several studies (Ruzzi and Aroca, 2015; Bona *et al*., 2016) have demonstrated that PGPR can increase the sweetness, moisture content, levels of secondary metabolites with antioxidant properties (such as anthocyanins, flavonoids and carotenoids) and contents of minerals in fruits included in the human diet (grapes, apples, strawberries, blackberries, sweet cherries, tomatoes). The enhanced contents of minerals and chlorophylls in cabbages supplied with PGPR (Bona *et al*., 2016) are very interesting because the majority of Brassicaceae do not associate with mycorrhizal fungi, the rhizospheric microorganisms frequently used in studies dealing with the improvement of food crop quality. PGPR can be applied by root dipping of seedlings, seed dipping, root inoculation or soil drench.

The use of beneficial fungi in agriculture and horticulture has increased markedly over the past two decades. *Trichoderma* spp., especially those species attracted by root‐derived sugar and exudates, are promising fungi for the development of sustainable agriculture because some strains are able to increase the tolerance of crops to biotic and abiotic stresses and improve plant growth and yield (López‐Bucio *et al*., 2015). These fungi release auxins, small peptides, volatiles and other active metabolites that promote root branching and nutrient uptake capacity into the rhizosphere. Although the majority of studies have focused on the positive effect of *Trichoderma* spp. on growth and yield (López‐Bucio *et al*., 2015), Molla *et al*. (2012) found increased levels of carotenoids and minerals in tomato fruits and Colla *et al*. (2015) reported enhanced chlorophyll contents in leaves of lettuce, which suggest that *Trichoderma* spp. may be applied to improve the quality of food crops. Moreover, these fungi are safe for humans, livestock and crop plants (López‐Bucio *et al*., 2015). *Piriformospora indica* is a biotrophic mutualistic root endosymbiont belonging to the order Sebacinales (Basidiomycota) capable of mimicking arbuscular mycorrhizal fungi (AMF). It can be cultivated axenically. It can colonize roots of a wide range of higher plants and induces disease resistance and stress tolerance. Several studies have demonstrated that *P. indica* can improve growth and yield in crop, horticultural and medicinal plants (Gill *et al*., 2016). In medicinal plants, *P. indica* also induced the production of secondary metabolites with beneficial effects on human health, which suggests that this fungus could also favour the accumulation of compounds with nutraceutical properties in edible parts of food crops.

The most widely studied beneficial fungi are AMF, which establish mutualistic associations with most crops and horticultural plants. AMF are considered as a promising tool for improving plant growth and yield, as well as for enhancing the tolerance of plants to biotic and abiotic stresses. There is also increasing evidence that these symbiotic fungi can enhance the nutritional quality of food crops. While the extraradical hyphae that extend from the root into the soil increase the uptake of water and minerals, the establishment of the intraradical fungal structures involves a continuous cellular and molecular dialogue between AMF and plants that includes the activation of the antioxidant, phenylpropanoid or carotenoid metabolic pathways. The recent review by Rouphael *et al*. (2015) summarizes the effects of AMF on crop tolerance to abiotic stresses, such as drought, salinity, nutrient deficiency, heavy metals and adverse soil pH, and also the role of AMF as enhancers of the nutraceutical value of horticultural products. However, the production of compounds beneficial for human health in the edible parts of crops has been investigated only in a limited number of plant species. Baslam *et al*. (2011a, 2013a) reported increased levels of carotenoids, soluble phenolics (including anthocyanins), tocopherol and minerals in leaves of lettuces associated with AMF and concluded that mycorrhizal symbiosis can overcome reductions in nutritional quality in glasshouse lettuces cultivated in inappropriate growing seasons (Baslam *et al*., 2013b). Results, however, were modulated by the variety or cultivar of lettuce and the species of AMF (Baslam *et al*., 2011a), phosphorus fertilization (Baslam *et al*., 2011b) and environmental parameters (Baslam and Goicoechea, 2012; Baslam *et al*., 2012). Increased levels of secondary metabolites with antioxidant properties have also been found in fruits of mycorrhizal strawberry plants (Castellanos‐Morales *et al*., 2010) and recent findings have shown that AMF inoculation of grapevines induced the accumulation of phenolics in berries and increased their antioxidant capacity under warm temperatures in the Tempranillo variety (Torres *et al*., 2016). Moreover, it has been reported that wines obtained from AMF‐inoculated plants had both a better oxidative stability and a significantly higher level of bioactive compounds compared with non‐mycorrhizal wines from Sangiovese variety (Gabriele *et al*., 2016). The ability of AMF to induce the accumulation of secondary metabolites with antioxidant properties is sometimes reinforced by the simultaneous incidence of some abiotic stresses. For example, mycorrhizal lettuces accumulated higher levels of anthocyanins in leaves when they were subjected to a moderate drought than under optimal irrigation (Baslam and Goicoechea, 2012). Likewise, the simultaneous application of AMF and water deficit favoured the accumulation of micronutrients and gliadins in grains of winter wheat (Goicoechea *et al*., 2016). This synergistic effect between water deficit and mycorrhizal symbiosis opens up the possibility of manipulating watering regimes in order to improve the nutritional quality of food crops, especially those cultivated in glasshouses. In contrast, the beneficial effect of AMF can disappear under elevated concentrations of CO_2_ in the atmosphere (Baslam *et al*., 2012; Goicoechea *et al*., 2016), which may represent a problem in future scenarios of climate change. Another factor that strongly influences the effectiveness of beneficial microorganisms for improving the nutritional quality of food crops is the coexistence of several microorganisms in the rhizosphere of a given host plant. Several studies have demonstrated that the inoculation of mixtures containing different species of AMF (Baslam *et al*., 2011a), AMF and PGPR (Lingua *et al*., 2013) or AMF and *Trichoderma* (Nzanza *et al*., 2012; Colla *et al*., 2015) enhances food crop quality in a higher degree than the individual application of these microorganisms.

## Concluding remarks and prospects

Increasing the nutritional value of food is possible by providing an appropriate rhizosphere microbiome for a given crop. However, there are several aspects that should be taken into account before the use of beneficial rhizospheric microorganisms is implemented in agriculture and horticulture. The effectiveness of the mutualistic associations will depend on the composition of the microbial communities, which is mainly determined by plant genotype and, to a lesser extent, by other biotic and abiotic factors (Patel *et al*., 2015). Moreover, different microorganisms can give different results in the same plant species (Garmendia *et al*., 2004; Bona *et al*., 2016); the same microorganism (or combination of microorganisms) can show different effectiveness even within cultivars of varieties belonging to the same plant species (Baslam *et al*., 2011a; Torres *et al*., 2016); and the effectiveness for a given mutualistic association is modulated by environmental factors (Baslam *et al*., 2012; Torres *et al*., 2016).

There can be also harmful aspects related to the application of potential beneficial microorganisms because some release compounds that exert a dual effect – positive and negative – on plant growth and development (Vejan *et al*., 2016), and presumably on food quality. As negative impacts have been found for particular species of microorganisms under certain specific conditions, the selection of the beneficial microorganisms appears once more to be a crucial aspect for achieving the best results in terms of crop development, yield and food quality and security.

Agricultural practices can benefit or impair the populations of rhizospheric microorganisms (Rouphael *et al*., 2015). In general terms, while organic horticulture increases both the amount and the diversity of rhizospheric microorganisms, soil tillage and the application of fungicides strongly reduce the presence of fungi in soils. Crop rotation can enhance the density and diversity of microorganisms depending on the species of plants chosen for this practice. Therefore, the use of beneficial microorganisms for improving the nutritional quality of food crops needs to be included in an integrated management programme in which the agricultural practices are of great importance.

Different strategies may be applied for enhancing the nutritional quality of food crops by using beneficial rhizospheric microorganisms: the manipulation of the crop microbiome *in situ* (Singh and Trivedi, 2017) and the external application of commercial inocula containing beneficial microorganisms in soils (Vosátka *et al*., 2012; Rouphael *et al*., 2015). The first approach requires the integration of emerging technologies (omics, for example) with traditional approaches of microbial ecology and plant ecophysiology. The second approach requires very close collaboration between the private sector and scientific community. Quality assurance is one of the main difficulties in the production of commercial inocula. High quality implies the production of non‐contaminated inocula formulated so that they can stored and distributed without losing viability and are easy to transport and to apply. The permanence of the inocula once introduced into agricultural soils over time and the possible impact on the indigenous microbioma should also be verified.

## Conflict of Interest

The authors assert no conflict of interest associated with this study.

## Acknowledgements

The authors wish to thank Paul William Miller for the language revision.
